# P-499. HIV Risk Perception, Substance Use, and Sexual Practices Among Circuit Party Attendees

**DOI:** 10.1093/ofid/ofae631.698

**Published:** 2025-01-29

**Authors:** Alex Dubov, Jennifer Veltman, Ethan Miles

**Affiliations:** Loma Linda University, Loma Linda, California; Loma Linda University School of Medicine, Linda Loma, California; Loma Linda University, Loma Linda, California

## Abstract

**Background:**

Circuit parties are large-scale dance events catering to the LGBTQ+ community. Research suggests high rates of HIV and substance use among participants. This study explores HIV knowledge, awareness of prevention services, perceived and actual HIV risk among attendees of the White Party, a circuit party held in California with an estimated 30,000 participants.

**Methods:**

Data were gathered from 137 attendees who visited a health booth, where interested individuals completed a self-administered survey on a tablet computer. The survey used the validated Minority AIDS Initiative (MAI) Adult Questionnaire assessing demographics, perceived risk related to substance use and sexual activities, and self-reported risk behaviors in these domains. Following survey completion, participants received a non-monetary incentive (event merchandise).

**Results:**

The average age of respondents was 38 years, with 26% identifying as Hispanic, 63% as White, 8% as Black, and 17% as Asian. Bivariate and multivariable logistic regression analyses were conducted to explore associations between knowledge of HIV status, awareness of HIV prevention resources, reports of unprotected sex, and the number of sexual partners. Younger participants and those with lower income levels were less likely to know their HIV status. Similarly, Hispanic respondents and those with lower income levels were less likely to know how to access HIV and SUD prevention. A higher perceived risk did not reduce a high incidence (82%) of unprotected sex. Individuals with a history of intimate partner violence (IPV) (18%) were more likely to engage in unprotected sex. Additionally, non-injection substance use was correlated with binge drinking and a higher number of sexual partners.

**Conclusion:**

This study highlights the need for comprehensive interventions at LGBTQ+ events, addressing substance use and HIV risk simultaneously. Interventions focusing on raising awareness of HIV risks may not be effective in changing behavior. Available interventions should include screening and support for IPV, prioritizing HIV testing among younger attendees, offering culturally tailored outreach in Spanish, and addressing socioeconomic barriers to accessing prevention services.

**Disclosures:**

**All Authors**: No reported disclosures

